# Improving Phosphorus Availability in an Acid Soil Using Organic Amendments Produced from Agroindustrial Wastes

**DOI:** 10.1155/2014/506356

**Published:** 2014-06-16

**Authors:** Huck Ywih Ch'ng, Osumanu Haruna Ahmed, Nik Muhamad Ab. Majid

**Affiliations:** ^1^Department of Crop Science, Faculty of Agriculture and Food Sciences, Universiti Putra Malaysia Bintulu Sarawak Campus, 97008 Bintulu, Sarawak, Malaysia; ^2^Institute of Tropical Forestry and Forest Products (INTROP), Universiti Putra Malaysia, 43400 Serdang, Selangor, Malaysia

## Abstract

In acid soils, soluble inorganic phosphorus is fixed by aluminium and iron. To overcome this problem, acid soils are limed to fix aluminium and iron but this practice is not economical. The practice is also not environmentally friendly. This study was conducted to improve phosphorus availability using organic amendments (biochar and compost produced from chicken litter and pineapple leaves, resp.) to fix aluminium and iron instead of phosphorus. Amending soil with biochar or compost or a mixture of biochar and compost increased total phosphorus, available phosphorus, inorganic phosphorus fractions (soluble inorganic phosphorus, aluminium bound inorganic phosphorus, iron bound inorganic phosphorus, redundant soluble inorganic phosphorus, and calcium bound phosphorus), and organic phosphorus. This was possible because the organic amendments increased soil pH and reduced exchangeable acidity, exchangeable aluminium, and exchangeable iron. The findings suggest that the organic amendments altered soil chemical properties in a way that enhanced the availability of phosphorus in this study. The amendments effectively fixed aluminium and iron instead of phosphorus, thus rendering phosphorus available by keeping the inorganic phosphorus in a bioavailable labile phosphorus pool for a longer period compared with application of Triple Superphosphate without organic amendments.

## 1. Introduction

Phosphorus is deficient in most acid soils because soluble inorganic P is fixed by Al and Fe [1]. This reaction contributes to less availability of P for crops. Information on the chemical forms of P is fundamental to the understanding of soil P dynamics and its interaction in acidic soils. This is necessary for the management of P in agriculture. The availability of P is influenced by soil organic matter, pH, and exchangeable and soluble Al, Fe, and Ca [2]. Phosphorus is generally available to crops at soil pH of 6 and 7. When the soil pH is less than 6, P deficiency increases in most crops. Conventionally, large amounts of lime and inorganic P fertilizers such as phosphate rocks and Triple Superphosphate (TSP) are used to saturate Al and Fe ions. This approach has not been successful because it is not economical. The practice is also not environmentally friendly. For example, overliming precipitates P ions with Ca as calcium phosphate, whereas excessive use of P fertilizers causes eutrophication. To date, phosphate rocks are acidulated to render P availability to crops.

In Malaysia, approximately 13 t ha^−1^ of pineapple (*Ananas comosus*) residues are produced on tropical peat soils per cropping season but the residues are commonly managed through burning [3]. Burning does not only cause haze and pollution but also causes peat fire. One of the challenges in the agroindustrial wastes management in Malaysia, Indonesia, and elsewhere is to add value to these wastes (e.g., converting pineapple residues into compost). Biochar is a carbonaceous substance produced as soil additive for agricultural and environmental management [4]. Increasing wastes disposal, global warming, and food insecurity call for the use of biochar (produced from agroindustrial wastes) in agriculture. This is essential because biochar improves soil fertility, crop productivity, soil water retention, and soil carbon sequestration [5]. Compost produced from pineapple leaves and biochar could be used to minimize P fixation in acid soils.

Although there exists some information on P sorption and fixation using organic matter [6, 7], there is a dearth of information on the use of biochar and compost to reduce P fixation. This is because these organic amendments have high affinity for Al and Fe. Their affinity enables long term chelation of Al and Fe by biochar and compost instead of P. Hence P will become readily and timely available for efficient crop use. Therefore, the objective of this study was to improve P availability by using biochar and compost produced from chicken litter and pineapple leaves, respectively, to fix Al and Fe instead of P.

## 2. Materials and Methods

An incubation study was carried out for 90 days at Universiti Putra Malaysia Bintulu Sarawak Campus. Bekenu Series (*Typic Paleudults*) soil at Universiti Putra Malaysia Bintulu Sarawak Campus which has not been cultivated was sampled at 0–20 cm. The sampling area was 50 m × 50 m, and 20 soil samples were randomly taken from this area. This soil was selected because it is commonly cultivated with different crops in Malaysia although the soil fixes P. The soil samples were air-dried, ground, and sieved to pass a 2 mm sieve after which they were bulked. From the bulked, 300 g of the soil was taken for each treatment into 500 mL beaker and each treatment was replicated three times. The rates of inorganic P fertilizer (TSP), pineapple leaves compost produced from our previous study [8], and commercially produced biochar (produced from chicken litter) were 60 kg P_2_O_5_ ha^−1^, 10 t ha^−1^, and 20 t ha^−1^, respectively. These rates were based on the standard recommendation for maize (*Zea mays* L.) cultivation [9–11]. The TSP and organic amendments requirements were scaled down to per 500 mL beaker. The treatments evaluated were as follows:

- soil only (T0),
- 300 g soil + 5.0 g TSP (T1),
- 300 g soil + 5.0 g TSP + 28.8 g biochar (T2),
- 300 g soil + 5.0 g TSP + 14.4 g compost (T3),
- 300 g soil + 5.0 g TSP + 14.4 g compost + 28.8 g biochar (T4).

The soil, TSP, biochar, and compost were thoroughly mixed. Beakers with the treatments were sealed with parafilm. The parafilm was perforated to enable good aeration. The treatments were arranged in a completely randomized design. The samples were incubated for 30 days, 60 days, and 90 days at 27°C, respectively. Each treatment had 3 replications (i.e., 15 samples for 30 days of incubation, 15 samples for 60 days of incubation, and 15 samples for 90 days of incubation). The soil samples were maintained at field capacity throughout the incubation study. At 30 days, 60 days, and 90 days of incubation (DAI), the soil samples were air-dried and analyzed, respectively.

### 2.1. Analyses of Soil and Organic Amendments

The soil samples were analysed for pH and electrical conductivity (EC) using pH meter and EC meter [12]. Soil texture was determined using the hydrometer method [13]. Total organic matter (OM) and total carbon (C) were determined using the combustion method [14]. Total N was determined using the micro-Kjeldahl method [15]. Total P and available P were extracted using the method described by Tan [16] after which the blue method [17] was used to determine them. Afterwards, C/N and C/P ratios were calculated. Potassium, Ca, Mg, Na, and Fe were determined using the ammonium acetate method [18]. Exchangeable acidity and Al were determined using the method described by Anderson and Ingram [19]. Inorganic P retained by Al, Fe, and Ca were fractionated after the soil samples were incubated. All of the incubated soil samples were analyzed for P fractions using Kuo [20] procedure. The P fractions were sequentially extracted on the basis of their relative solubilities.

The biochar and compost were analysed for pH, EC, total OM, total C, and total N using the methods previously cited. Single dry ashing method [21] was used to extract P, K, Ca, Mg, Na, Zn, Cu, Fe, and Al in the biochar and compost. The filtrates were analyzed for K, Ca, Mg, Na, Zn, Cu, Fe, and Al using Atomic Absorption Spectrophotometer (AAS), whereas P was determined using the blue method [17].

### 2.2. Statistical Analysis

The study was a factorial experiment in completely randomized design (CRD) with two factors, namely, organic amendments (biochar and compost) and time of incubation (30 days, 60 days, and 90 days). Statistical analysis of data including analysis of variance (ANOVA) and comparison of means was performed using Statistical Analysis System (SAS) version 9.2. ANOVA was used to detect treatment effects while Tukey's test was used to compare treatment means at *P* ≤ 0.05.

## 3. Results and Discussion

### 3.1. Characteristics of Soil and Organic Amendments

The selected physicochemical properties of Bekenu Series (*Typic Paleudults*) (Table 1) are consistent with those reported in soil survey staff [22]. The pH, EC, C, N, P, K, Ca, Zn, Al, and Cu of the biochar were higher but the Mg and Na of the biochar were lower than those of compost (Table 2). The C/N ratios of the biochar and compost were 22.75 and 19.91, respectively, whereas their C/P ratios were 24.50 and 99.56, respectively. These ratios suggest net mineralization of the organic amendments. 

### 3.2. Effect of Organic Amendments on Soil pH, Exchangeable Acidity, Exchangeable Aluminium, Exchangeable Iron, and Exchangeable Calcium

Days of incubation significantly affected soil pH at 30 DAI, 60 DAI, and 90 DAI (Table 3). Soil pH as affected by treatments is summarized in Table 4. At 30 DAI, 60 DAI, and 90 DAI, the organic amendments (T2, T3, and T4) significantly increased soil pH compared with the nonorganic amendments (T0 and T1). The increase in soil pH was due to the rapid proton (H^+^) exchange between the soil and the organic amendments [23, 24].

The reduction in exchangeable acidity, exchangeable Al, and exchangeable Fe partly relates to the increase in soil pH (Table 4). Increase in pH resulted in the precipitation of exchangeable and soluble Al and Fe as insoluble Al and Fe hydroxides, thus reducing the concentrations of Al and Fe in the soil solution [25]. T2 and T4 showed reduced exchangeable Fe in the soil solution at 60 DAI and 90 DAI compared to T3. This finding relates to the initial content of Fe in the compost. The soil with TSP alone (T1) was higher in exchangeable Fe because the TSP may have released Fe into the soil. The organic amendments (T2, T3, and T4) increased exchangeable Ca in the soil solution compared with the nonorganic amendments (T0 and T1). This was due to the relatively high Ca content in the biochar and compost.

### 3.3. Effects of Organic Amendments on Total and Available Phosphorus

There were significant differences in the soil total P and available P at 30 DAI, 60 DAI, and 90 DAI (Table 5). All of the organic amendments (T2, T3, and T4) increased total and available P levels compared with soil alone (T0) and soil and TSP only (T1) at 30 DAI, 60 DAI, and 90 DAI (Table 6). The increasing availability of P with days of incubation contradicts the findings of several studies where a decline in available P with time was ascribed to P sorption [26, 27]. However, the P availability reported in this study is comparable to those reported by Laboski and Lamb [28], Spychaj-Fabisiak et al. [29], and Opala et al. [30]. The increase in the availability of P with time was because of microbially mediated mineralization of soil organic P to form inorganic P.

### 3.4. Effects of Organic Amendments on Phosphorus Fractions

There were significant differences of the soil P fractions at 30 DAI, 60 DAI, and 90 DAI (Table 5). Soluble-P is readily soluble in soil solution for plant uptake. It is also closely linked to the dynamics of P bounding in soil. It represents a nonspecific adsorption and ligand exchange on mineral edges. The organic amendments (T2, T3, and T4) increased soluble-P, Al-P, Fe-P, redundant soluble-P, and Ca-P compared with soil alone (T0) and soil and TSP alone (T1) (Table 6). This observation is consistent with that of Lee et al. [31] who also observed a significant increase in Al-P and Fe-P fractions upon application of organic amendments and inorganic fertilizers. In this study, Al-P was the dominant P fraction. This was followed by Ca-P, Fe-P, and redundant soluble-P. Calcium induced P sorption or precipitation because the organic amendments increased the soil Ca-P fraction. The increase in Ca-P fraction could also be associated with the chemistry and retention of Ca rather than the hydrolytic reaction of Al [32]. 

Al-P and Fe-P are available fractions for crops in acidic soils. This is contrary to redundant soluble-P which is occluded in acid soils. This process renders redundant soluble-P unavailable for crops. In acid soils, the original, superficial, loosely bound phosphates (Al and Fe oxides which are available to plants) are reprecipitated into highly crystalline Al-P and Fe-P (not available to crops) but the biochar and compost were able to fix Al and Fe. The sorption is essential for P availability because sorption reduces Al and Fe oxides in acid soils. The biochar and compost were also able to especially fix Al-P and Fe-P to prevent them from being further precipitated into sorbed P forms. Al-P and Fe-P fractions are biologically labile because increasing soil pH causes dissolution of Al-P and Fe-P to release P. The organic amendments (T2, T3, and T4) increased organic P (P_o_) compared with soil alone (T0) or soil and TSP alone (T1) (Table 6). At 90 DAI, the concentrations of P_o_ were relatively higher than in 30 DAI and 60 DAI. The increase in P_o_ with the increasing of time is essential as P_o_ will mineralize to release P into the soil solution for crop use.

## 4. Conclusion

Amending acid soil with biochar or compost or a mixture of biochar and compost increased total P, available P, inorganic P fractions (soluble-P, Al-P, Fe-P, redundant soluble-P, and Ca-P), and organic P. This was possible because the organic amendments increased soil pH, and, at the same time, they reduced exchangeable acidity, exchangeable Al, and exchangeable Fe. As the soil pH increased, the organic amendments effectively fixed Al and Fe instead of P. The findings suggest that the organic amendments altered soil chemical properties in a way that enhanced the availability of P in this study. The findings of this study are being evaluated in both green house and field experiments.

## Figures and Tables

**Table 1:** Selected physicochemical properties of Bekenu series soil.

Property	Value obtained
Bulk density (g cm^−3^)	1.23
Soil texture	Sand: 67.5%
	Silt: 15.5%
	Clay: 17.0%
	⇒ Sandy loam
pH (Water)	4.56
Total organic matter (%)	7.2
Total carbon (%)	4.18
Total N (%)	0.18
Total P (ppm)	132.30
Available P (ppm)	4.50
C/N ratio	23.2
C/P ratio	321.54
Cation exchange capacity (cmol_c_ kg^−1^)	5.1
Exchangeable acidity (cmol_c_ kg^−1^)	1.16
Exchangeable Al (cmol_c_ kg^−1^)	0.84
Exchangeable K (ppm)	1.16
Exchangeable Ca (ppm)	470.30
Exchangeable Mg (ppm)	553.00
Exchangeable Fe (ppm)	2300.00

**Table 2:** Selected chemical properties of chicken litter biochar and pineapple residue compost.

Property	Pineapple residue compost	Chicken litter biochar
pH	7.89	8.50
Electrical conductivity (dS m^−1^)	6.90	15.50
Total carbon (%)	45.80	63.70
Total N (%)	2.30	2.80
Total P (%)	0.46	2.60
C/N ratio	19.91	22.75
C/P ratio	99.56	24.50
Total K (%)	2.67	3.90
Total Ca (%)	0.40	5.90
Total Mg (g kg^−1^)	6365.0	15.20
Total Na (g kg^−1^)	1143.0	19.50
Total Zn (mg kg^−1^)	119.0	856.0
Total Cu (mg kg^−1^)	47.20	167.0
Total Fe (mg kg^−1^)	5062.0	2650.0
Total Al (mg kg^−1^)	1.50	0.60

**Table 3:** Mean square values of analysis of variance (ANOVA) to evaluate the effects of treatments and time on the soil pH, exchangeable acidity, exchangeable Al, exchangeable Fe, and exchangeable Ca.

Source of variations	Degree of freedom	Mean square				
		pH	Exchangeable acidity	Exchangeable Al	Exchangeable Fe	Exchangeable Ca
Treatments	4	3.71∗	0.23∗	0.08∗	9.03∗	70.70∗
Time	2	0.65∗	0.15∗	0.01∗	0.37∗	0.52∗
Treatments ∗ time	8	0.76∗	0.34∗	0.01∗	0.14∗	0.24∗
Error	30					

Note: *indicates significant at *P* ≤ 0.05.

**Table 4:** Effects of organic amendments and incubation time on the soil pH, exchangeable acidity, exchangeable Al, exchangeable Fe, and exchangeable Ca.

Treatments	pH	Exchangeable acidity	Exchangeable Al	Exchangeable Fe	Exchangeable Ca
		cmol kg^−1^			
30 DAI					
T0	5.09 ± 0.5^e^	0.67 ± 0.1^a^	0.22 ± 0.05^a^	0.3 ± 0.1^e^	0.03 ± 0.01^e^
T1	5.34 ± 0.3^d^	0.34 ± 0.1^c^	0.1 ± 0.02^b^	2.27 ± 0.2^b^	4.35 ± 0.2^d^
T2	5.69 ± 0.5^c^	0.41 ± 0.1^b^	Trace	1.14 ± 0.2^c^	6.21 ± 0.2^b^
T3	6.18 ± 0.5^b^	0.19 ± 0.05^d^	Trace	2.67 ± 0.25^a^	5.28 ± 0.2^c^
T4	6.68 ± 0.5^a^	0.19 ± 0.06^d^	Trace	0.96 ± 0.2^d^	7.15 ± 0.3^a^
60 DAI					
T0	4.29 ± 0.5^e^	0.9 ± 0.1^a^	0.22 ± 0.05^a^	0.26 ± 0.1^e^	0.03 ± 0.1^e^
T1	5.63 ± 0.4^d^	0.37 ± 0.1^c^	0.06 ± 0.02^b^	2.26 ± 0.2^b^	4.13 ± 0.2^d^
T2	5.87 ± 0.5^c^	0.29 ± 0.1^b^	Trace	0.21 ± 0.2^c^	6.3 ± 0.2^b^
T3	6.24 ± 0.4^b^	0.19 ± 0.05^d^	Trace	2.35 ± 0.3^a^	5.22 ± 0.2^c^
T4	6.69 ± 0.4^a^	0.22 ± 0.05^d^	Trace	0.96 ± 0.2^d^	6.61 ± 0.3^a^
90 DAI					
T0	4.33 ± 0.5^e^	0.89 ± 0.1^a^	0.22 ± 0.05^a^	0.25 ± 0.1^e^	0.03 ± 0.1^e^
T1	5.54 ± 0.4^d^	0.37 ± 0.1^c^	0.05 ± 0.02^b^	2.22 ± 0.2^b^	4.17 ± 0.2^d^
T2	6.79 ± 0.5^c^	0.25 ± 0.1^b^	Trace	0.23 ± 0.2^c^	6.46 ± 0.2^b^
T3	6.35 ± 0.3^b^	0.19 ± 0.04^d^	Trace	2.33 ± 0.3^a^	5.63 ± 0.3^c^
T4	6.63 ± 0.3^a^	0.16 ± 0.04^d^	Trace	0.89 ± 0.2^d^	6.45 ± 0.3^a^

Means within column with different letter(s) indicate significant difference between treatments by Tukey's test at *P* ≤ 0.05.

**Table 5:** Mean square values of analysis of variance (ANOVA) to evaluate the effects of treatments and time on the soil P fractions.

Source of variations	Degree of freedom	Mean square						
		Total P	Available P	Al-P	Fe-P	Redundant soluble-P	Ca-P	Total organic P
Treatments	4	11312939.81∗	13753397.87∗	500115.64∗	104348.54∗	28800.00∗	144318.64∗	3686913.29∗
Time	2	843661.83∗	555927.70∗	74138.99∗	50139.27∗	9790.33∗	55006.27∗	55006.27∗
Treatments ∗ time	8	145199.71∗	468613.19∗	78580.74∗	7944.11∗	2171.50∗	9244.67∗	9244.67∗
Error	30							

Note: *indicates significant at *P* ≤ 0.05.

**Table 6:** Effects of organic amendments and incubation time on the soil total P, available P, soluble-P, Al-P, Fe-P, redundant soluble-P, Ca-P, and total organic P.

Treatments	Total P	Available P	Soluble-P	Al-P	Fe-P	Redundant soluble-P	Ca-P	Total organic P
	ppm							
30 DAI								
T0	134.2 ± 26^e^	40.49 ± 12^e^	8.05 ± 2^e^	16.1 ± 4^e^	12.08 ± 3^e^	0.8 ± 0.2^e^	3.23 ± 1.5^e^	93.94 ± 10^e^
T1	3265.6 ± 325^d^	816.4 ± 55^d^	196 ± 38^d^	392 ± 40^d^	294 ± 44^d^	19.6 ± 2^d^	78.4 ± 5^d^	2285.6 ± 210^d^
T2	4555 ± 432^b^	1595 ± 234^b^	273.4 ± 45^b^	546.8 ± 48^b^	410.1 ± 42^b^	27.3 ± 3^b^	109.36 ± 6^b^	3188 ± 160^b^
T3	3550 ± 450^c^	1038.5 ± 340^c^	213 ± 38^c^	426 ± 44^c^	319 ± 43^c^	21.3 ± 3^c^	85.2 ± 5^c^	2485 ± 210^c^
T4	5015 ± 470^a^	3290 ± 370^a^	301 ± 42^a^	602 ± 53^a^	415.5 ± 45^a^	30.1 ± 3^a^	120.4 ± 10^a^	3513 ± 180^a^
60 DAI								
T0	133.2 ± 32^e^	40.74 ± 13^e^	8.1 ± 3^e^	16 ± 5^e^	12 ± 3^e^	0.8 ± 0.2^e^	3.06 ± 1.5^e^	93.24 ± 10^e^
T1	3371 ± 360^d^	842.75 ± 59^d^	202.3 ± 39^d^	405.6 ± 48^d^	303.446 ± 46^d^	21.3 ± 2^d^	78.7 ± 4^d^	2395.7 ± 210^d^
T2	4617 ± 445^b^	1615.95 ± 241^b^	278 ± 8^b^	555 ± 44^b^	415.5 ± 44^b^	28.7 ± 3^b^	107.9 ± 6^b^	3231 ± 180^b^
T3	3650 ± 452^c^	1131.5 ± 352^c^	220 ± 41^c^	440 ± 48^c^	329 ± 45^c^	22 ± 3^c^	84 ± 6^c^	2555 ± 240^c^
T4	5049 ± 455^a^	3231.4 ± 380^a^	303.2 ± 44^a^	607 ± 45^a^	454 ± 45^a^	30.3 ± 3^a^	120.7 ± 10^a^	3533 ± 190^a^
90 DAI								
T0	136.6 ± 33^e^	40.9 ± 16^e^	8 ± 3^e^	16.05 ± 5^e^	12.1 ± 4^e^	0.8 ± 0.2^e^	3.15 ± 1.5^e^	96.5 ± 13^e^
T1	3465.5 ± 350^d^	866.38 ± 64^d^	207.94 ± 41^d^	416 ± 40^d^	311.9 ± 48^d^	21.78 ± 2^d^	82.08 ± 4^d^	2425.8 ± 230^d^
T2	4713 ± 440^b^	1885.2 ± 252^b^	264.1 ± 52^b^	528 ± 48^b^	396.2 ± 47^b^	28.4 ± 3^b^	103.8 ± 7^b^	3392.4 ± 190^b^
T3	3895 ± 468^c^	1363.25 ± 360^c^	218.2 ± 43^c^	435.4 ± 42^c^	327 ± 47^c^	22 ± 4^c^	87.4 ± 5^c^	2804 ± 250^c^
T4	5143 ± 467^a^	3342.95 ± 390^a^	288 ± 47^a^	576 ± 48^a^	432 ± 40^a^	30.8 ± 4^a^	113.2 ± 10^a^	3702.9 ± 210^a^

Means within column with different letter(s) indicate significant difference between treatments by Tukey's test at *P* ≤ 0.05.
